# Supplementary material for: Validating daily social media macroscopes of emotions
Source: Sci Rep. 2022 Jul 4;12:11236. doi: 10.1038/s41598-022-14579-y (PMC9253324; doi:10.1038/s41598-022-14579-y)
Supplement: Supplementary file 1 — Supplementary Information. [file 41598_2022_14579_MOESM1_ESM.pdf]

Supplementary Information to 'Validating daily social media macroscopes of emotions'

Max Pellert,[1,2,3,4*] Hannah Metzler,[2,3,4] Michael Matzenberger,[5] David Garcia[2,3,4]

[1]Sony Computer Science Laboratories,
6, Rue Amyot, 75005 Paris, France
[2]Institute of Interactive Systems and Data Science,
Graz University of Technology,
Inffeldgasse 16C, 8010 Graz, Austria
[3]Complexity Science Hub Vienna,
Josefstädter Straße 39, 1080 Vienna, Austria
[4]Section for Science of Complex Systems, Center for Medical Statistics,
Informatics and Intelligent Systems, Medical University of Vienna,
Spitalgasse 23, 1090 Vienna, Austria
[5]Der Standard,
Vordere Zollamtsstraße 13, 1030 Vienna, Austria

*To whom correspondence should be addressed; E-mail: pellert@csh.ac.at.

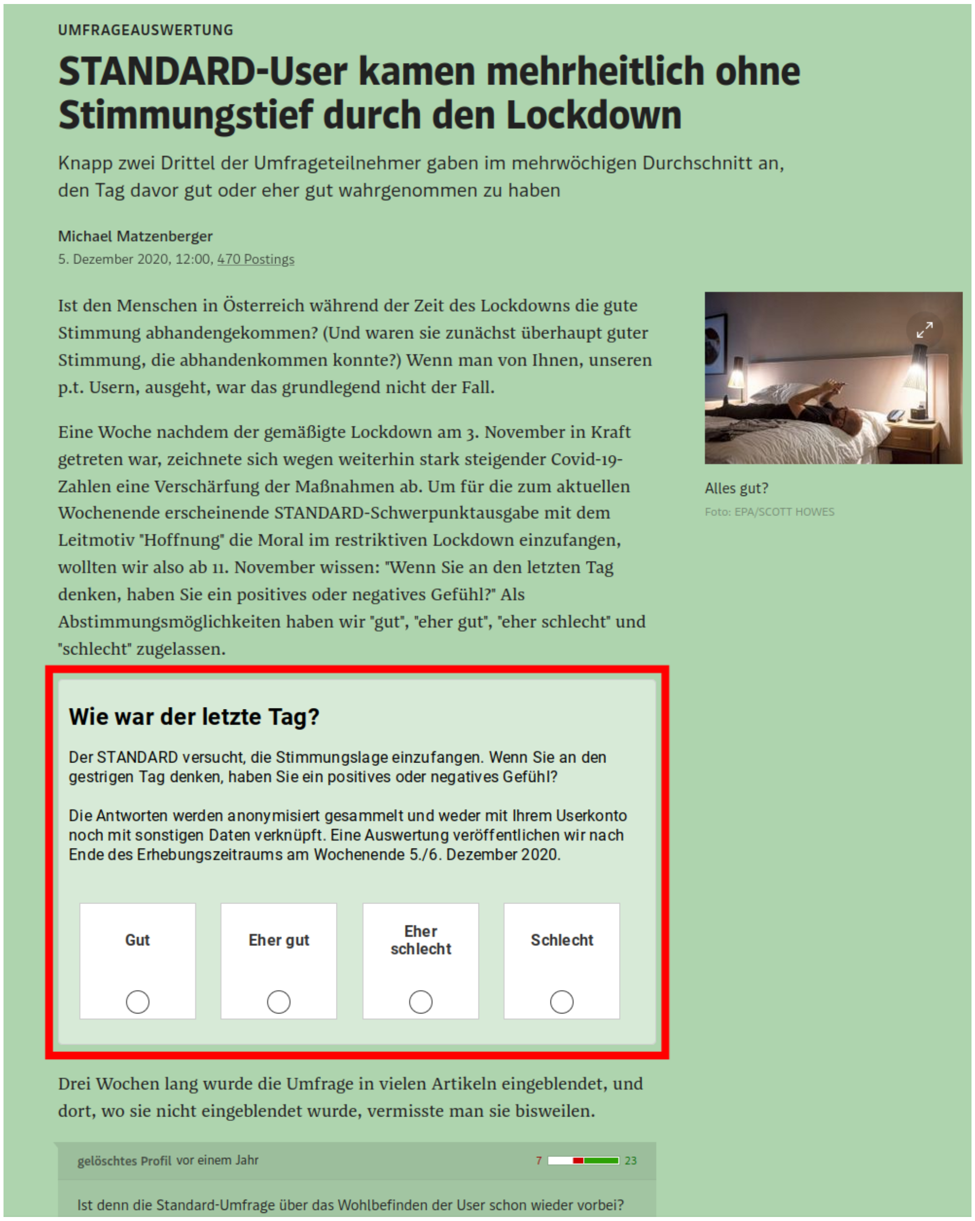

UMFRAGEAUSWERTUNG

STANDARD-User kamen mehrheitlich ohne Stimmungstief durch den Lockdown

Knapp zwei Drittel der Umfrageteilnehmer gaben im mehrwöchigen Durchschnitt an, den Tag davor gut oder eher gut wahrgenommen zu haben

Michael Matzenberger
5. Dezember 2020, 12:00, 470 Postings

Ist den Menschen in Österreich während der Zeit des Lockdowns die gute Stimmung abhandengekommen? (Und waren sie zunächst überhaupt guter Stimmung, die abhandenkommen konnte?) Wenn man von Ihnen, unseren p.t. Usern, ausgeht, war das grundlegend nicht der Fall.

Alles gut?
Foto: EPA/SCOTT HOWES

Eine Woche nachdem der gemäßigte Lockdown am 3. November in Kraft getreten war, zeichnete sich wegen weiterhin stark steigender Covid-19-Zahlen eine Verschärfung der Maßnahmen ab. Um für die zum aktuellen Wochenende erscheinende STANDARD-Schwerpunktausgabe mit dem Leitmotiv "Hoffnung" die Moral im restriktiven Lockdown einzufangen, wollten wir also ab 11. November wissen: "Wenn Sie an den letzten Tag denken, haben Sie ein positives oder negatives Gefühl?" Als Abstimmungsmöglichkeiten haben wir "gut", "eher gut", "eher schlecht" und "schlecht" zugelassen.

Wie war der letzte Tag?

Der STANDARD versucht, die Stimmungslage einzufangen. Wenn Sie an den gestrigen Tag denken, haben Sie ein positives oder negatives Gefühl?

Die Antworten werden anonymisiert gesammelt und weder mit Ihrem Userkonto noch mit sonstigen Daten verknüpft. Eine Auswertung veröffentlichen wir nach Ende des Erhebungszeitraums am Wochenende 5./6. Dezember 2020.

Gut | Eher gut | Eher schlecht | Schlecht

Drei Wochen lang wurde die Umfrage in vielen Artikeln eingeblendet, und dort, wo sie nicht eingeblendet wurde, vermisste man sie bisweilen.

gelöschtes Profil vor einem Jahr 7 23

Ist denn die Standard-Umfrage über das Wohlbefinden der User schon wieder vorbei?

Figure S1: Screenshot of derstandard.at showing a news article where the survey (red rectangle added by the authors) is displayed in between the article text. The full text of the survey contains the following information (translated to English by the authors): "How was the last day? Der Standard tries to capture general mood. If you think about yesterday, do you have a positive or negative feeling? Answers are collected anonymously and not linked to your user account, nor any other data. The results will be published at the end of the survey period on the weekend of December 5/6 2020."

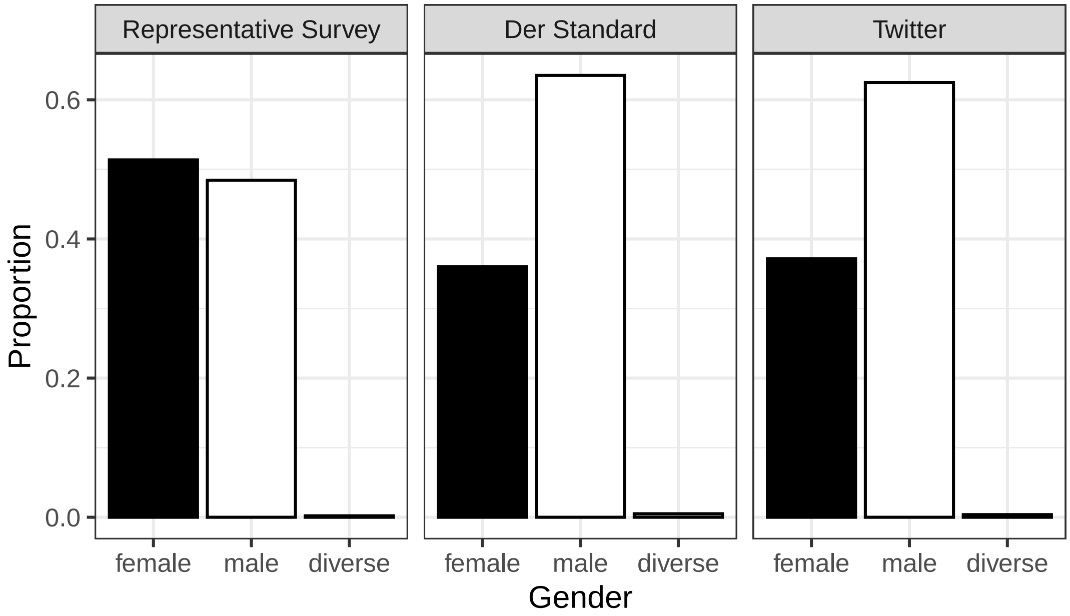

Figure S2: Males are over-represented both on Der Standard and on Twitter, and to a similar extent.

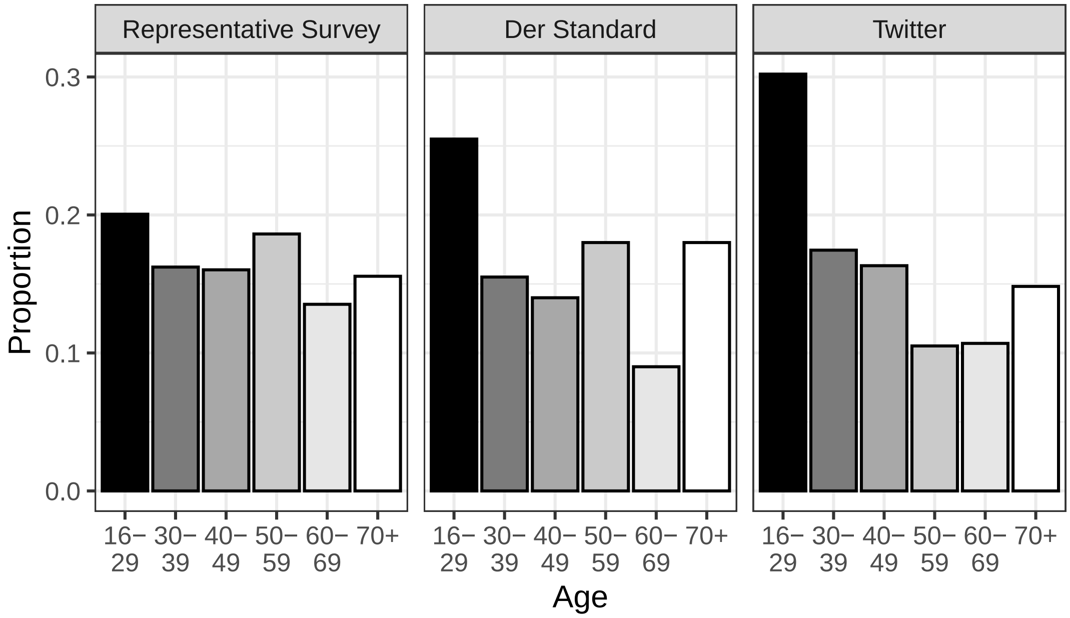

Figure S3: The youngest cohort (16-29) is over-represented on Der Standard and even more so on Twitter. 50-59 year-olds are under-represented on Twitter only, whereas 60-69 year-olds are under-represented on both platforms.

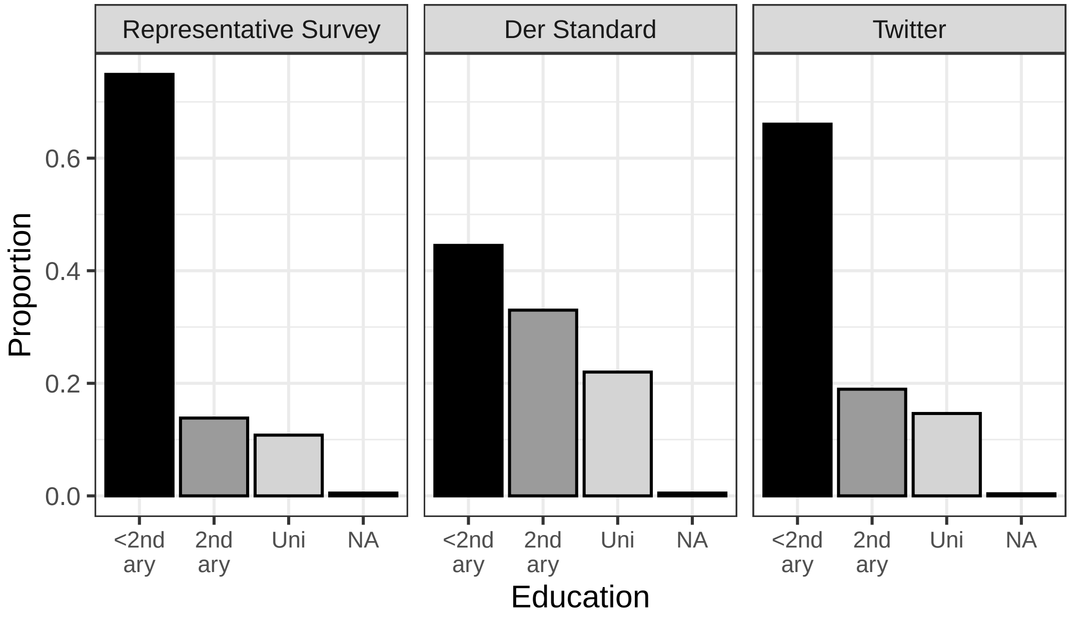

Figure S4: Users with education below secondary school (”Matura” in Austria) are under-represented, especially on Der Standard, whereas higher education is over-represented.

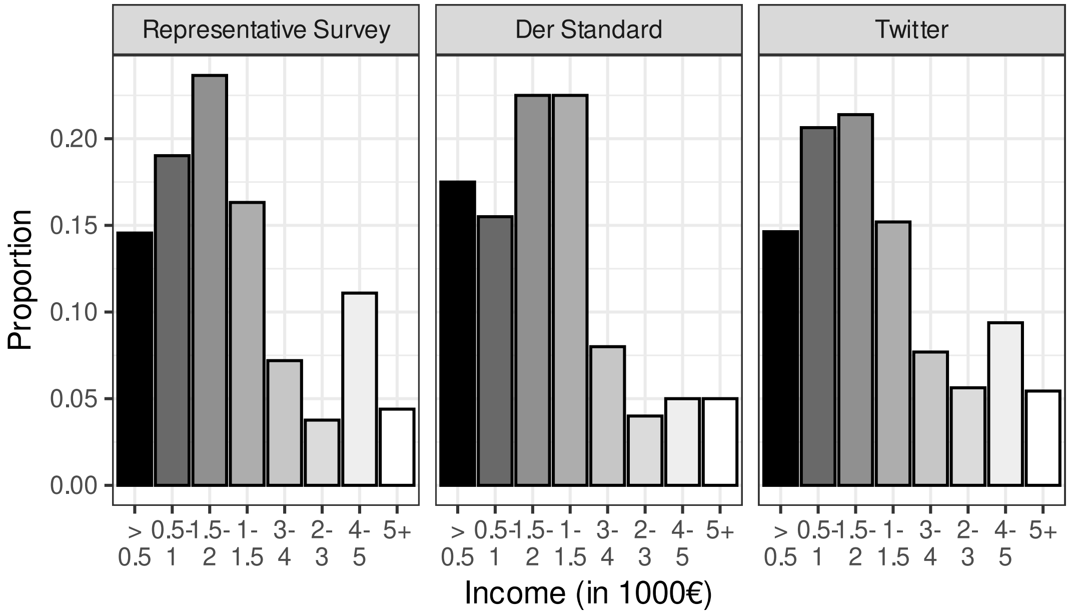

Figure S5: Self-reported income does not differ strongly, although some categories might be slightly over- or underrepresented.

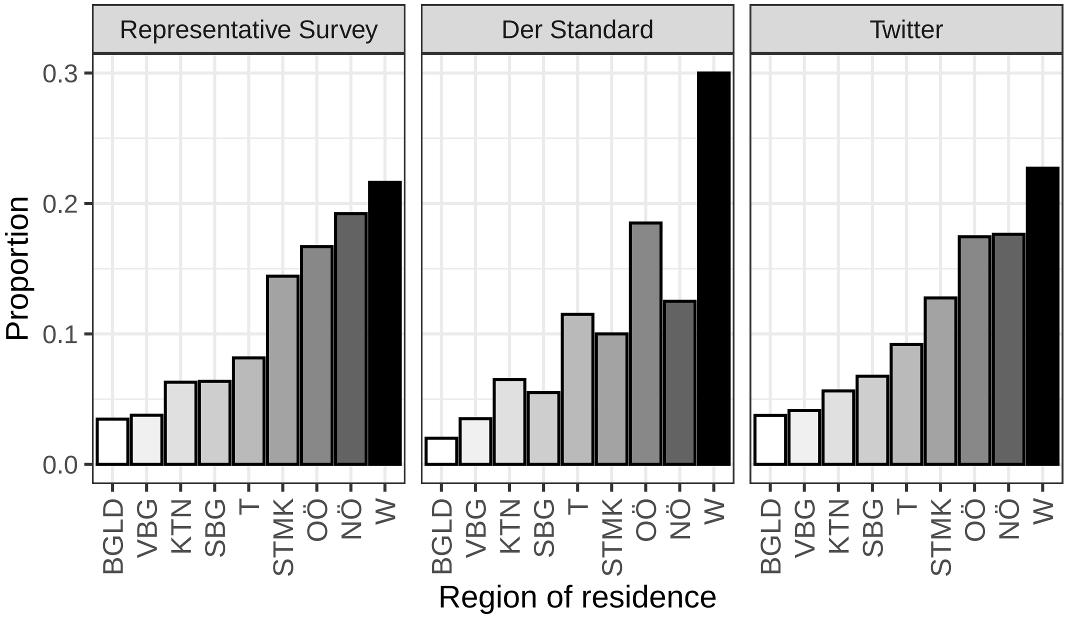

Figure S6: Region of residence is close to representative on Twitter. On Der Standard, Vienna and Upper Austria (”OÖ”) are over-represented, while Styria (”STMK”) and Lower Austria (”NÖ”) are under-represented.)

Table S1 shows that for Der Standard, correlations of LIWC and aggregate sentiment with COVID-19 cases are significantly lower compared to the survey. In contrast, the correlation of GS sentiment alone is not significantly different compared to the survey. For Twitter, none of the correlations with COVID-19 cases differs between the sentiment measures and the survey. Overall, the signal from the survey is thus not more correlated with new cases than Twitter sentiment. Yet, when using data from Der Standard, only one of the sentiment methods (GS) correlates as strongly with cases as the survey, suggesting that the LIWC dictionary could be improved for this particular data source.

Table S1: Significance tests comparing correlations with the number of new COVID-19 cases between aggregate sentiment on both platforms and the survey. We test the difference in correlations of sentiment measures with new COVID-19 cases compared to the survey with new COVID-19 cases.

	p value	Difference (higher)
Twitter (Aggregate Sentiment) vs. Survey	0.501	0.06 (Twitter)
Twitter (LIWC) vs. Survey	0.617	0.05 (Twitter LIWC)
Twitter (GS) vs. Survey	0.644	0.04 (Twitter GS)
Der Standard (Aggregate Sentiment) vs. Survey	0.015	-0.2 (Survey)
Der Standard (LIWC) vs. Survey	0.023	-0.36 (Survey)
Der Standard (GS) vs. Survey	0.087	-0.16 (Survey)

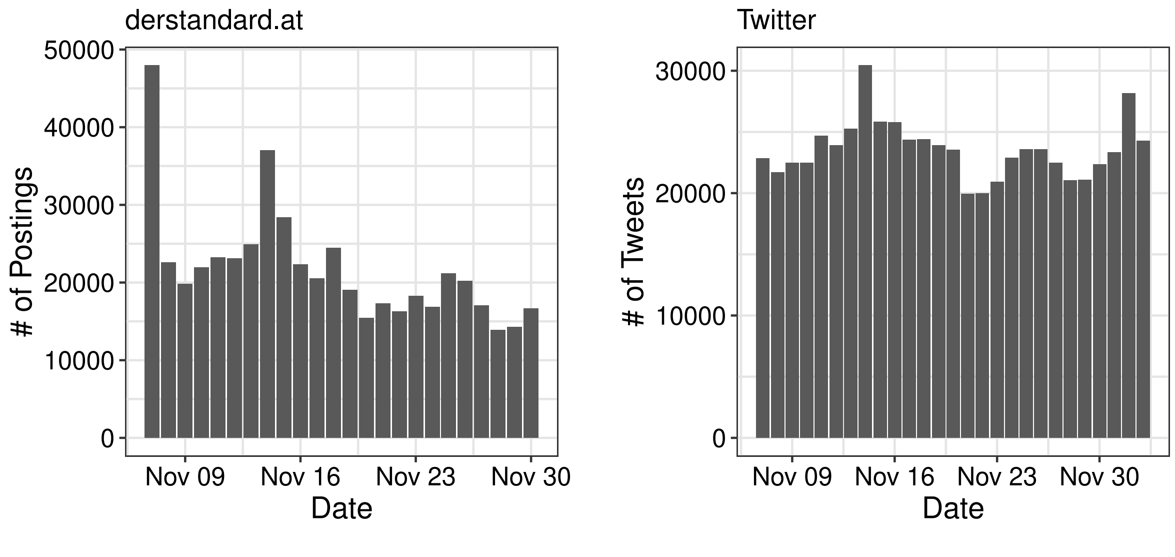

Figure S7: Number of posts per day for both platforms.

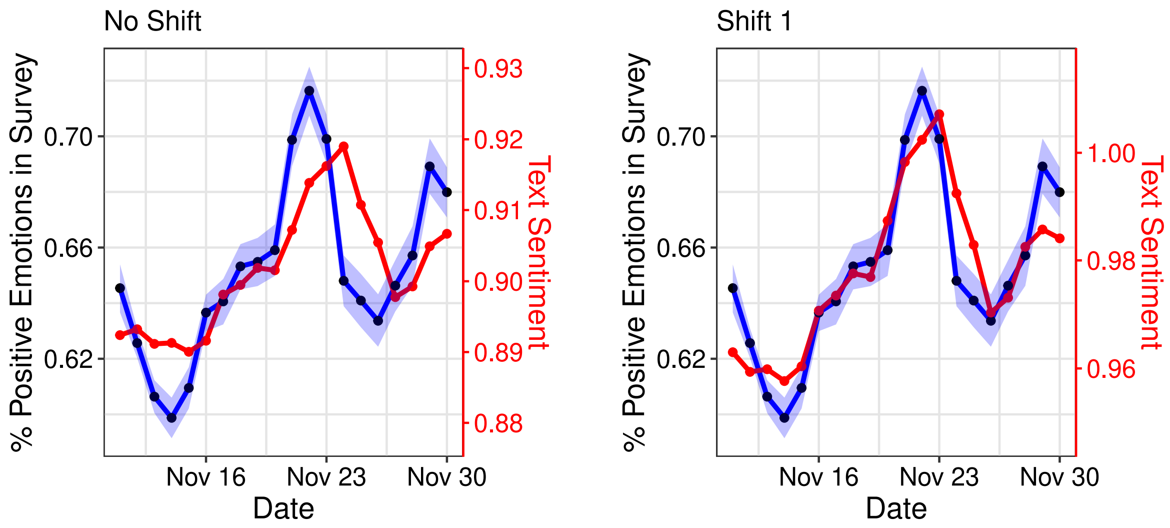

Figure S8: Twitter is slightly delayed compared to postings on derstandard.at postings. A shift of one day corrects the slower response on Twitter.

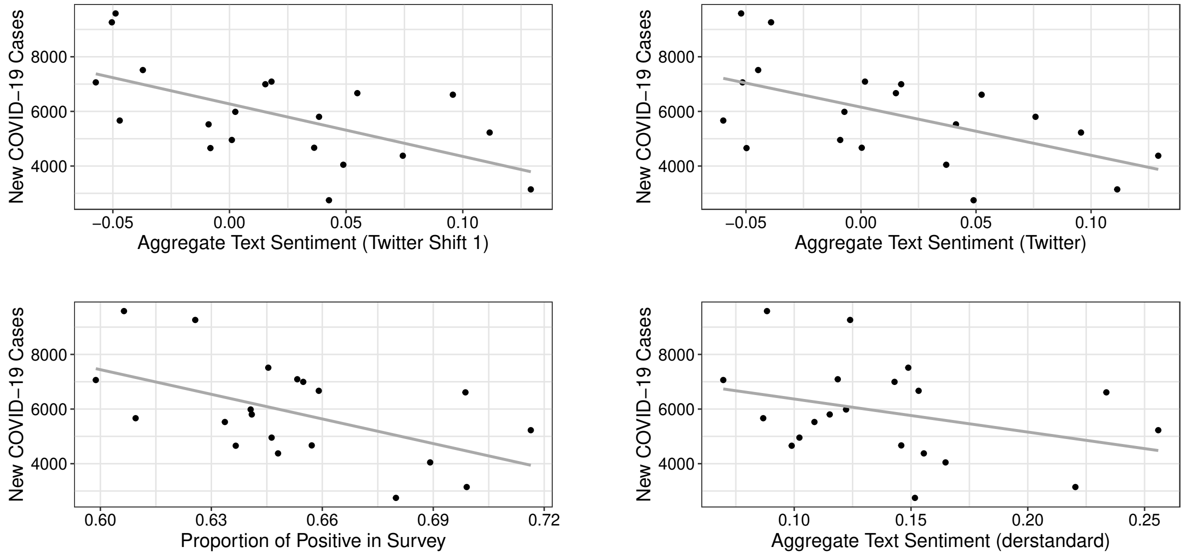

Figure S9: Scatterplots for aggregate text sentiment and positive survey responses (see Table 3 in the main document) vs. the number of new COVID-19 cases in Austria.

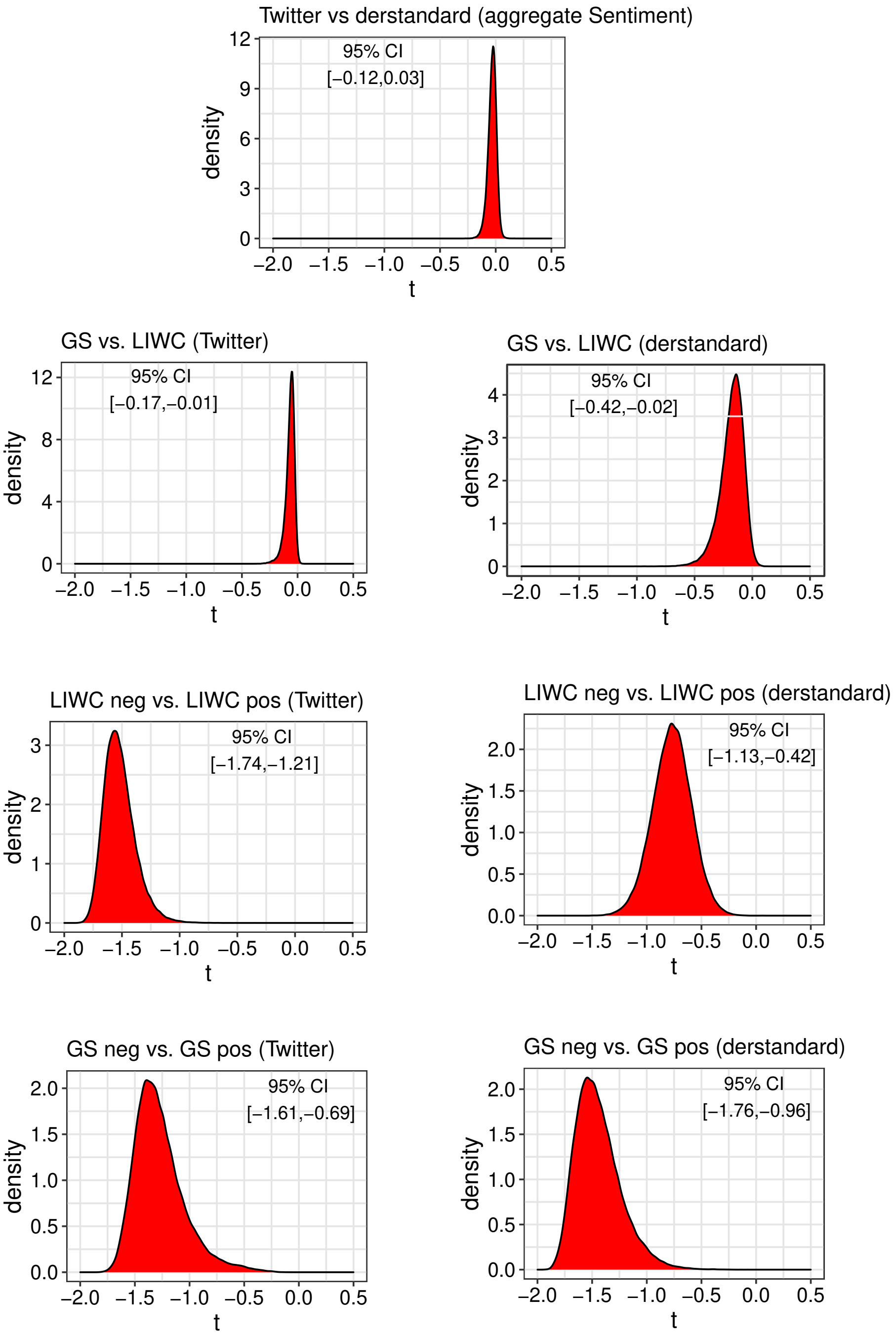

Figure S10: Results of bootstrapping the differences between two correlation coefficients (Table 1 in the main document) for 100 000 times.

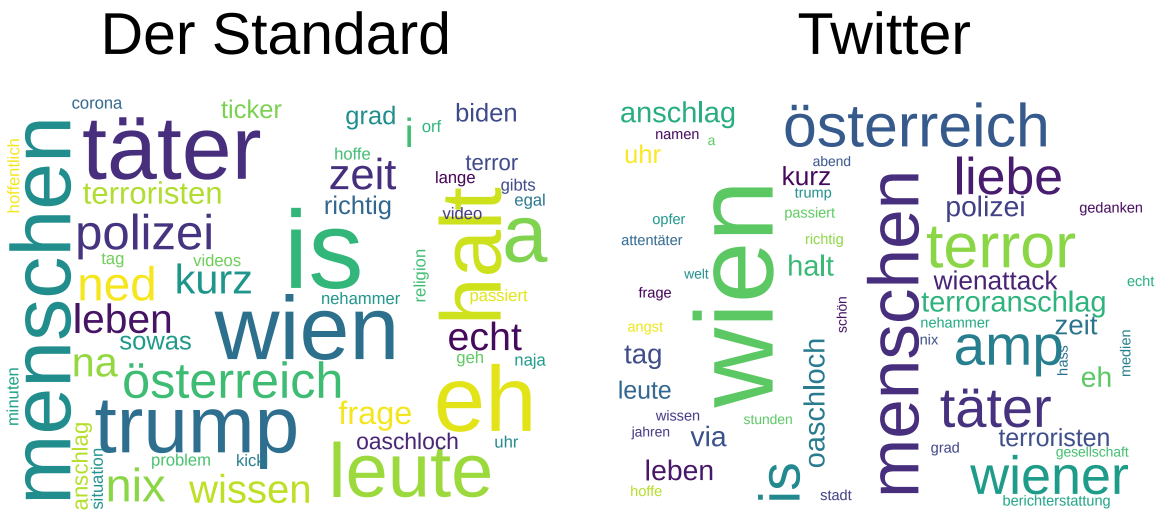

Figure S11: Word clouds of the text on both platforms on 2020-11-03, the day after a terrorist attack in Vienna. We see references to ”Täter”/perpetrator and location (”Wien”/Vienna), ”Menschen”/”people” on both platforms. We display the 40 most common words and filter stop words using the full list of German stop words from `https://github.com/solariz/german_stopwords`.

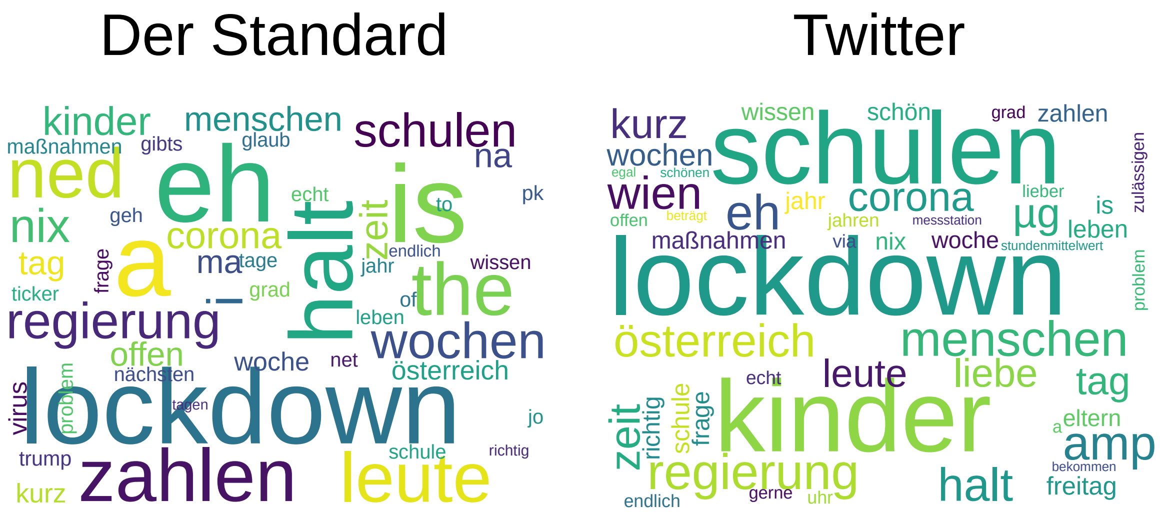

Figure S12: Word clouds of the text on both platforms on 2020-11-13, the day of the highest increase of cases in the second COVID-19 wave in Austria. References to ”lockdown”, ”kinder”/”children” and ”schulen”/”schools” reflect similar discussions on both platforms. We display the 40 most common words and filter stop words using the full list of German stop words from `https://github.com/solariz/german_stopwords`.

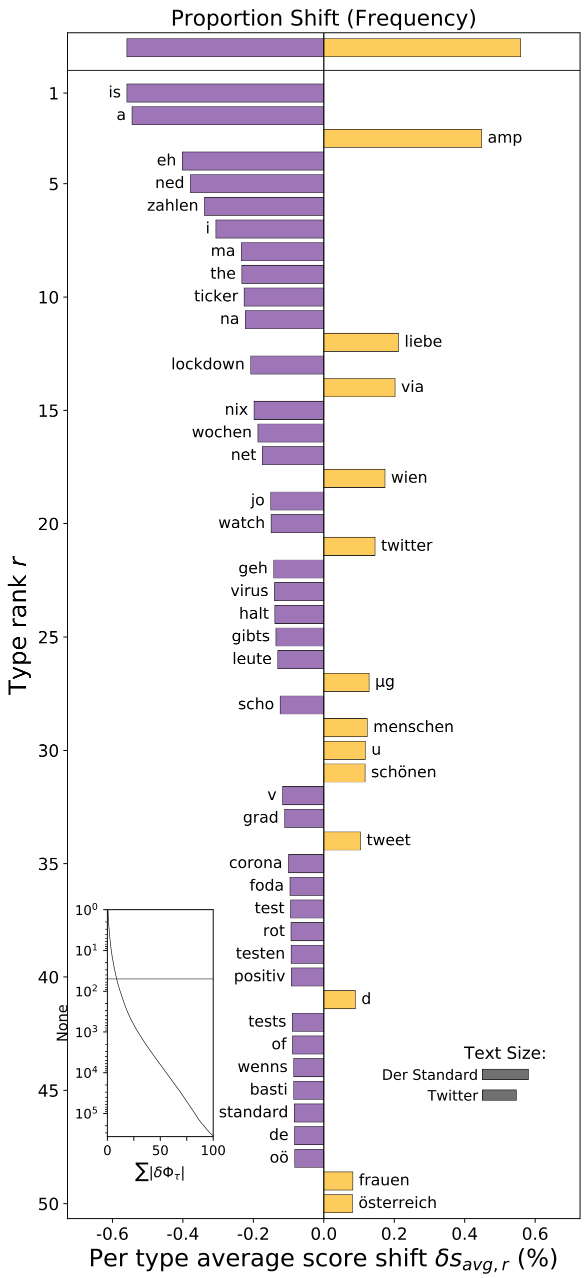

Figure S13: Wordshift graph comparing the text of both platforms during the survey period from 2020-11-11 - 2020-11-30. The shift shows Twitter minus Der Standard. The purple (yellow) bars show words that are more frequent on Der Standard (Twitter). The biggest differences in the corpora are trivial dialect words of Austrian German on Der Standard. Apart from that, there seem to be more direct mentions of COVID-19 related concepts (virus, test, lockdown, test) on Der Standard. We filter stop words using the full list of German stop words from `https://github.com/solariz/german_stopwords`.
